# Characteristics and Correlates of Cigarette Smoking Status Among US Adults Receiving Federal Housing Assistance

**DOI:** 10.5888/pcd15.170395

**Published:** 2018-04-26

**Authors:** Teresa W. Wang, Veronica E. Helms, Peter J. Ashley, Brian A. King

**Affiliations:** 1 Office on Smoking and Health, National Center for Chronic Disease Prevention and Health Promotion, Centers for Disease Control and Prevention, Atlanta, Georgia; 2 Epidemic Intelligence Service, Centers for Disease Control and Prevention, Atlanta, Georgia; 3 Office of Research, Evaluation, and Monitoring, Office of Policy Development and Research, US Department of Housing and Urban Development, Washington, DC; 4 Office of Lead Hazard Control and Healthy Homes, US Department of Housing and Urban Development, Washington, DC

## Abstract

This study describes patterns of cigarette smoking (current, former, never) by sociodemographic, household, and chronic disease characteristics and correlates among US adults receiving housing assistance from the US Department of Housing and Urban Development (HUD) during 2007–2012. Estimates were generated from 4,771 adults by using National Health Interview Survey and HUD-linked data. Overall, 48.4% of HUD-assisted adults were never smokers, 33.0% were current smokers, and 18.6% were former smokers; smoking status varied by sex, age, race/ethnicity, whether children were living in the household, and chronic disease status. These estimates could inform tobacco control interventions to improve the health and well-being of HUD-assisted residents.

## Objective

Assisted housing is an important platform for improving the health and well-being of residents through evidence-based tobacco control interventions (1). Nearly 10 million people, including 4 million children, receive housing assistance from the US Department of Housing and Urban Development (HUD); approximately 2 million of these people reside in public housing (2). Effective February 2017, HUD finalized a rule requiring all public housing agencies to prohibit the use of lit tobacco products in and around their properties within 18 months (3). To provide data for informing policy implementation, the objective of this study was to describe characteristics and correlates of cigarette smoking status (current, former, never) among HUD-assisted adults.

## Methods

The National Center for Health Statistics (NCHS) linked HUD administrative data to data from respondents of the National Health Interview Survey (NHIS), a cross-sectional household survey conducted by using multistage area probability design to capture a statistically representative sample of the civilian, noninstitutionalized US population (1,4). The NCHS Research Ethics Review Board approved the linkage of the data. During 2007–2012, 4,771 of 4,788 total linked respondents aged 18 or older completed the NHIS sample adult module, reported information about their smoking status, and resided in HUD-assisted rental housing (multifamily, housing choice voucher, or public housing) at the time of the NHIS interview.

Smoking status was assessed by asking, “Have you smoked at least 100 cigarettes in your entire life?” and “Do you now smoke cigarettes every day, some days, or not at all?” Current smokers reported smoking at least 100 lifetime cigarettes and now smoking every day or some days; former smokers reported smoking at least 100 lifetime cigarettes and now smoking not at all; never smokers reported not smoking at least 100 lifetime cigarettes.

Data were weighted to account for the complex survey design, and weights were adjusted for linkage eligibility by using a model-based calibration approach (4). The percentage distribution of HUD-assisted current, former, and never smokers was stratified by sex (male or female), age (18–44 y, 45–64 y, or ≥65 y), race/ethnicity (Hispanic, non-Hispanic white, non-Hispanic black, or other non-Hispanic racial/ethnic minority groups), presence of other people living in the household (yes or no), presence of children aged 17 years or younger living in the household (yes or no), and number of diagnosed chronic conditions (0, 1, or ≥2). Estimates from sample sizes less than 30 were suppressed. All potential pairwise comparisons were conducted for each demographic construct by using 2-sided *t* tests, with *P* < .05 indicating significance. For constructs with more than 2 comparisons (ie, age, race/ethnicity, chronic conditions), Bonferroni adjustments were implemented to account for multiple hypothesis testing.

## Results

During 2007–2012, 48.4% of HUD-assisted adults were never smokers, 33.0% were current smokers, and 18.6% were former smokers (Table 1). The percentage of never smokers was significantly higher among adults aged 18 to 44 (54.0%) or 65 years or older (49.9%) than among adults aged 45 to 64 (37.6%); women (51.1%) than men (41.0%); Hispanic adults (58.8%) and non-Hispanic black adults (54.6%) than non-Hispanic white adults (36.8%); those who did not live alone (51.5%) than those who lived alone (43.3%); and those who lived with at least 1 child (51.2%) than those who did not live with children (46.0%). The percentage of former smokers was significantly higher among adults aged 65 years or older (35.0%) than among the other 2 age groups; men (23.9%) than women (16.7%); non-Hispanic white adults (24.5%) than Hispanic adults (16.8%) or non-Hispanic black adults (13.6%); those who lived alone (26.9%) than those who did not live alone (13.5%); and those who did not live with children (24.2%) than those who lived with at least 1 child (12.0%). The percentage of current smokers was significantly higher among those aged 45 to 64 years (41.1%) than among the other 2 age groups; non-Hispanic white adults (38.7%) than non-Hispanic black adults (31.8%) and Hispanic adults (20.0%); those who did not live alone (35.0%) than among those who lived alone (29.9%); and those who lived with at least 1 child (36.8%) than among those who did not live with children (29.8%).

**Table 1:** Cigarette Smoking Status Among HUD-Assisted Adults, by Sociodemographic and Housing Characteristics, NHIS-HUD Linked Data, 2007–2012

Characteristic	Never Smokers^a^		Former Smokers^b^		Current Smokers^c^	
	n	% (95% CI)	n	% (95% CI)	n	% (95% CI)
**Overall**	2,292	48.4 (46.0–50.8)	944	18.6 (17.1–20.2)	1,535	33.0 (30.8–35.3)
**Sex**						
Male	417	41.0 (36.5–45.7)	309	23.9 (20.8–27.4)	398	35.1 (30.9–39.6)
Female	1,875	51.1 (48.6–53.6)	635	16.7 (15.1–18.4)	1,137	32.3 (29.9–34.8)
**Age, y**						
18–44	1,183	54.0 (50.7–57.3)	204	10.2 (8.6–12.0)	773	35.8 (32.8–38.9)
45–64	546	37.6 (34.3–41.1)	315	21.3 (18.5–24.3)	588	41.1 (37.7–44.5)
≥65	563	49.9 (45.8–54.0)	425	35.0 (31.4–38.6)	174	15.1 (12.3–18.3)
**Race/ethnicity**						
Hispanic	536	58.8 (54.6–62.8)	150	16.8 (13.8–20.0)	206	20.0 (20.7–28.6)
Non-Hispanic white	575	36.8 (33.1–40.6)	419	24.5 (21.7–27.6)	597	38.7 (34.9–42.6)
Non-Hispanic black	1,080	54.6 (51.7–57.6)	335	13.6 (11.9–15.4)	675	31.8 (29.0–34.7)
Non-Hispanic other **d**	101	47.7 (31.3–64.5)	40	19.6 (13.0–27.7)	57	32.7 (21.3–45.9)
**Presence of others in household^e^**						
Lives alone	977	43.3 (40.5–46.0)	631	26.9 (24.8–29.1)	659	29.9 (27.0–32.8)
Does not live alone	1,315	51.5 (48.2–54.8)	313	13.5 (11.7–15.5)	876	35.0 (32.1–37.9)
**Living with children^e^**						
≥1 Child aged 0–17 y	1,071	51.2 (47.6–54.7)	219	12.0 (9.9–14.4)	740	36.8 (33.6–40.1)
No children aged 0–17 y	1,221	46.0 (43.3–48.7)	725	24.2 (22.3–26.2)	795	29.8 (27.3–32.4)

Abbreviations: CI, confidence interval; HUD, US Department of Housing and Urban Development; NHIS, National Health Interview Survey.

a Participants were classified as never smokers if they responded no to the question, “Have you smoked at least 100 cigarettes in your entire life?” and “not at all” to the question, “Do you now smoke cigarettes every day, some days, or not at all?”

b Participants were classified as former smokers if they responded yes to the question, “Have you smoked at least 100 cigarettes in your entire life?” and responded with “not at all” to the question, “Do you now smoke cigarettes every day, some days or not at all?”

c Participants were classified as current smokers if they responded yes to the question, “Have you smoked at least 100 cigarettes in your entire life?” and responded with “every day,” or “some days” to the question, “Do you now smoke cigarettes every day, some days or not at all?”

d Includes non-Hispanic Asian and all other non-Hispanic racial groups.

e These data were collected in the NHIS family core module.

Smoking status also varied by the number of chronic conditions among HUD-assisted adults (Table 2). Overall, the percentage of never smokers was significantly higher among those without chronic conditions (59.6%) than among those with 1 chronic condition (49.1%) or 2 or more chronic conditions (39.2%). In contrast, the percentage of former smokers was higher among those with 2 or more chronic conditions (27.2%) than among those with 1 chronic condition (15.4%) or no chronic conditions (9.9%). After stratification by age, chronic disease status significantly differed by current, former, and never smokers aged 18 to 44 years and never smokers aged 65 or older.

**Table 2:** Cigarette Smoking Status Among HUD-Assisted Adults, by Chronic Conditions^a^ and Age, NHIS-HUD Linked Data, 2007–2012

No. of Chronic Conditions^a^	Never Smokers^b^		Former Smokers^c^		Current Smokers^d^	
	n	% (95% CI)	n	% (95% CI)	n	% (95% CI)
**Overall**						
0	856	59.6 (55.9–63.3)	149	9.9 (8.1–11.9)	459	30.5 (27.2–34.0)
1	545	49.1 (44.9–53.3)	196	15.4 (12.9–18.3)	372	35.5 (31.4–39.7)
≥2	891	39.2 (36.2–42.3)	599	27.2 (24.5–29.9)	704	33.6 (30.7–36.6)
**18–44 y**						
0	703	62.7 (58.7–66.5)	84	7.6 (5.7–10.0)	358	29.7 (26.2–33.4)
1	319	49.2 (43.5–55.0)	72	11.5 (8.3–15.2)	238	39.3 (34.0–44.9)
≥2	161	35.6 (28.3–43.6)	48	16.0 (7.0–29.4)	177	48.4 (40.8–56.0)
**45–64 y**						
0	104	44.8 (36.0–53.8)	40	16.4 (11.3–22.6)	88	38.8 (30.4–47.7)
1	113	40.6 (33.7–47.8)	59	17.5 (12.5–23.5)	114	41.9 (35.0–49.1)
≥2	329	34.6 (30.4–39.1)	216	23.9 (19.9–28.3)	386	41.5 (37.3–45.8)
**≥65 y**						
0	49	56.9 (42.9–70.0)	25	—e	13	—e
1	113	61.9 (53.1–70.1)	65	28.7 (21.5–36.7)	20	—e
≥2	401	46.5 (42.1–51.0)	335	37.2 (33.1–41.3)	141	16.3 (13.0–20.2)

Abbreviations: CI, confidence interval; HUD, US Department of Housing and Urban Development; NHIS, National Health Interview Survey.

a Participants were identified as having a chronic condition if they reported in the survey that they had ever been told by a doctor or health care provider that they had hypertension, coronary heart disease, stroke, diabetes, cancer, arthritis, or hepatitis; had experienced weak or failing kidneys during the past 12 months; currently had asthma; or had chronic obstructive pulmonary disease (COPD). COPD was assessed using affirmative responses from 2 survey questions asking adults if they had ever had emphysema or had had chronic bronchitis in the past 12 months; adults who responded yes to either question were identified as having COPD. These data were collected in the sample adult questionnaire.

b Participants were classified as never smokers if they responded no to the question, “Have you smoked at least 100 cigarettes in your entire life?” and “not at all” to the question, “Do you now smoke cigarettes every day, some days, or not at all?”

c Participants were classified as former smokers if they responded yes to the question, “Have you smoked at least 100 cigarettes in your entire life?” and responded with “not at all” to the question, “Do you now smoke cigarettes every day, some days or not at all?”

d Participants were classified as current smokers if they responded yes to the question, “Have you smoked at least 100 cigarettes in your entire life?” and responded with “every day,” or “some days” to the question, “Do you now smoke cigarettes every day, some days or not at all?”

e Estimate suppressed as nominal sample size was less than 30.

## Discussion

Before release of the linked NHIS–HUD data, no data sources existed to provide national estimates of smoking status among HUD-assisted residents. This study complements a previous report on current smoking and health outcomes among HUD-assisted adults (1) by providing more nuanced data on smoking status among this population, most notably data on former and never smokers. During 2007–2012, nearly half of HUD-assisted residents were never smokers, while approximately one-fifth were former smokers. These data along with variations observed by sociodemographic, household, and chronic disease characteristics can help inform educational efforts and shifts in social norms that prevent the initiation of tobacco product use, promote cessation, and reduce the likelihood of relapse.

This study indicates that former and never smokers comprise the majority (67%) of HUD-assisted residents. These people are susceptible to secondhand smoke exposure in their homes; approximately one-third of multiunit housing residents who prohibit smoking in their personal living units experience secondhand smoke incursions from elsewhere in their building (5–7). These incursions are a public health concern because there is no risk-free level of second-hand smoke exposure (8). Moreover, smoking in multiunit housing imposes considerable financial costs. The implementation of smoke-free US-subsidized housing has been projected to yield annual cost savings of nearly $500 million from averted health care costs, reduced renovation costs in units where smoking has occurred, and reduced smoking-attributable fire losses (9).

Our study has limitations. First, approximately 60% of 2007–2012 NHIS adults were linkage eligible, which may introduce selection bias if those who were linkage-eligible differed from those who were not linkage-eligible. However, linkage-adjusted weights were used to account for differences between these groups by sex, age, and race/ethnicity (4). Second, all data were based on self-report. Third, these cross-sectional data did not capture data on smoking status for the same cohort as they aged. Finally, this analysis assumed that the composition of HUD-assisted adults has not significantly changed over time.

These data can help inform the development, implementation, and sustainment of evidence-based strategies (10), including smoke-free policies, to reduce the burden of tobacco use–related death and disease among HUD-assisted children and adults. Continued surveillance of smoking and health behaviors among populations living in HUD-assisted housing is important to further evaluate the impact and outcome of these strategies.
